# Architecture and subunit arrangement of the complete *Saccharomyces cerevisiae* COMPASS complex

**DOI:** 10.1038/s41598-018-35609-8

**Published:** 2018-11-27

**Authors:** Yanxing Wang, Zhanyu Ding, Xiangyang Liu, Yu Bao, Min Huang, Catherine C. L. Wong, Xiaoyu Hong, Yao Cong

**Affiliations:** 1 National Center for Protein Science Shanghai, State Key Laboratory of Molecular Biology, CAS Center for Excellence in Molecular Cell Science, Shanghai Institute of Biochemistry and Cell Biology, Chinese Academy of Sciences, University of Chinese Academy of Sciences, Shanghai, 201210 China; 2 Shanghai Science Research Center, Chinese Academy of Sciences, Shanghai, 201210 China; 3 Center for Precision Medicine Multi-Omics Research, Peking University Health Science Center, State Key Laboratory of Natural and Biomimetic Drugs, School of Pharmaceutical Sciences, Peking University, Beijing, 100191 China

## Abstract

Methylation of histone H3 lysine 4 (H3K4) is catalyzed by the multi-component COMPASS or COMPASS-like complex, which is highly conserved from yeast to human, and plays essential roles in gene expression and transcription, cell cycle progression, and DNA repair. Here we present a cryo-EM map of the complete *S*. *cerevisiae* COMPASS complex. Through tag or Fab labeling strategy combined with cryo-EM 3D reconstruction and cross-linking and mass spectrometry (XL-MS) analysis, we uncovered new information on the subunit arrangement: Cps50, Cps35, and Cps30 were determined to group together to form the face region in the head of the complex, and Cps40 and the N-terminal portion of Set1 reside on the top of the head. Our map reveals the location of the active center and a canyon in the back of the head. Together, our study provides the first snapshot of the complete architecture of yeast COMPASS and a picture of its subunit interaction network, which could facilitate our understanding of the COMPASS machinery and its functionality.

## Introduction

Methylation of histone H3 lysine 4 (H3K4) is required for the epigenetic maintenance of transcriptionally active forms of chromatin in eukaryotes^1,2^. H3K4 can be mono-, di-, and tri-methylated, and the methylation is catalyzed by the SET domain-containing enzymes^3^. While most SET domain-containing proteins can on their own act as histone methyltransferases, the MLL/Set1 family methyltransferases, which catalyze histone H3K4 methylation, must form multi-protein complexes for maximal catalytic and biological activities^2^. Such a complex was first purified in budding yeast and named COMPASS (complex of proteins associated with Set1)^4^. Only one type of COMPASS has been identified in yeast, while in human the COMPASS family is divided into six members, including SET1A, SET1B, and MLL1-MLL4^5^. COMPASS has been demonstrated from yeast to human to be a fundamentally and evolutionarily conserved family of enzymes and to be a central regulator of gene expression. Consequently, perturbation of its composition and activities can alter normal biological processes in development, including cell proliferation and differentiation^6^.

The yeast COMPASS complex consists of seven distinct subunits, including Set1, Cps60/Bre2, Cps50/Swd1, Cps40/Spp1, Cps35/Swd2, Cps30/Swd3, and Cps25/Sdc1^4^. Moreover, Cps15/Shg1 was identified as an additional subunit of yeast COMPASS, however, no mammalian homologs of Cps15 has been identified thus far and the loss of this subunit has no effect on COMPASS stability or functionality^3^. Set1 alone in yeast is inactive, but within COMPASS complex it can perform the mono-, di-, and tri-methylation of H3K4^5^, indicating each subunit has a specific function in the regulation of H3K4 methylation, Set1 stability, or COMPASS assembly^2,7^. Cps50 and Cps30, two WD40 repeat-containing proteins, can stably associate with each other to form a heterodimer and are required for the integrity of the complex, which is critical for maintaining global levels of H3K4 methylation^7^. Cps60 and Cps40 are required for achieving proper levels of di- and tri-methylation of H3K4^8^. Cps60 shares high sequence homology with drosophila Ash2 and human ASH2L, and is a member of the trithorax family of homeodomain DNA-binding proteins^4^. Moreover, Cps60 was found to form a heterodimer with Cps25^3^, one of the smallest subunits of the complex. Cps40 and the n-SET domain (762–937) of Set1 are required for the stability of Set1^9^. Cps35, another WD40 repeat-containing protein, is essential in budding yeast^10,11^ and is required for maintaining proper levels of H3K4me2 and H3K4me3^5,8,9,12^. Many of these findings for yeast COMPASS complex also hold true for human COMPASS family. Different modules of COMPASS are involved in distinct functions, including COMPASS assembly, regulation of substrate recognition and H3K4 methylation, and cofactor binding. It is possible that, to fulfill the multiple functionalities of different modules, this multi-component molecular machine might be intrinsically dynamic.

To identify the structural elements underpinning COMPASS assembly, several crystal structures of its key components and subcomplexes have been determined^13–22^, which provided information about its subunit interaction network. In addition, extensive efforts have been made to reconstitute fully functional yeast COMPASS and human COMPASS-like complexes *in vitro* to identify the minimum subunit composition required for histone H3K4 methylation^7,9,23^. A 24-Å-resolution map of the core complex of yeast COMPASS was produced using cryo-EM, and this core complex showed a Y-shaped architecture consisting of Cps50, Cps30, the C-terminal SET domain of Set1, Cps60, and Cps25^7^. However, due to the lack of the complete structure of COMPASS, the entire spectrum of interactions controlling COMPASS assembly remains unclear, which hinders our understanding of the mechanisms underlying its substrate recognition and H3K4 methylation.

To obtain a thorough picture of the COMPASS machinery, we overexpressed and purified the complete and functionally active *Saccharomyces cerevisiae* COMPASS complex in budding yeast. We present a cryo-EM map of this complete yeast COMPASS complex at 10.0 Å resolution. By combining subunit-specific eGFP^24,25^, DID^26^, or PA–NZ-1^27–29^ labeling strategy with cryo-EM 3D reconstruction, as well as cross-linking and mass spectrometry (XL-MS) analysis, we provide a complete picture of the subunit organization and full molecular architecture of yeast COMPASS. This study could facilitate our understanding of the COMPASS machinery and its functionality.

## Results

### Yeast COMPASS overexpression, purification, and activity validation

**Figure 1:**
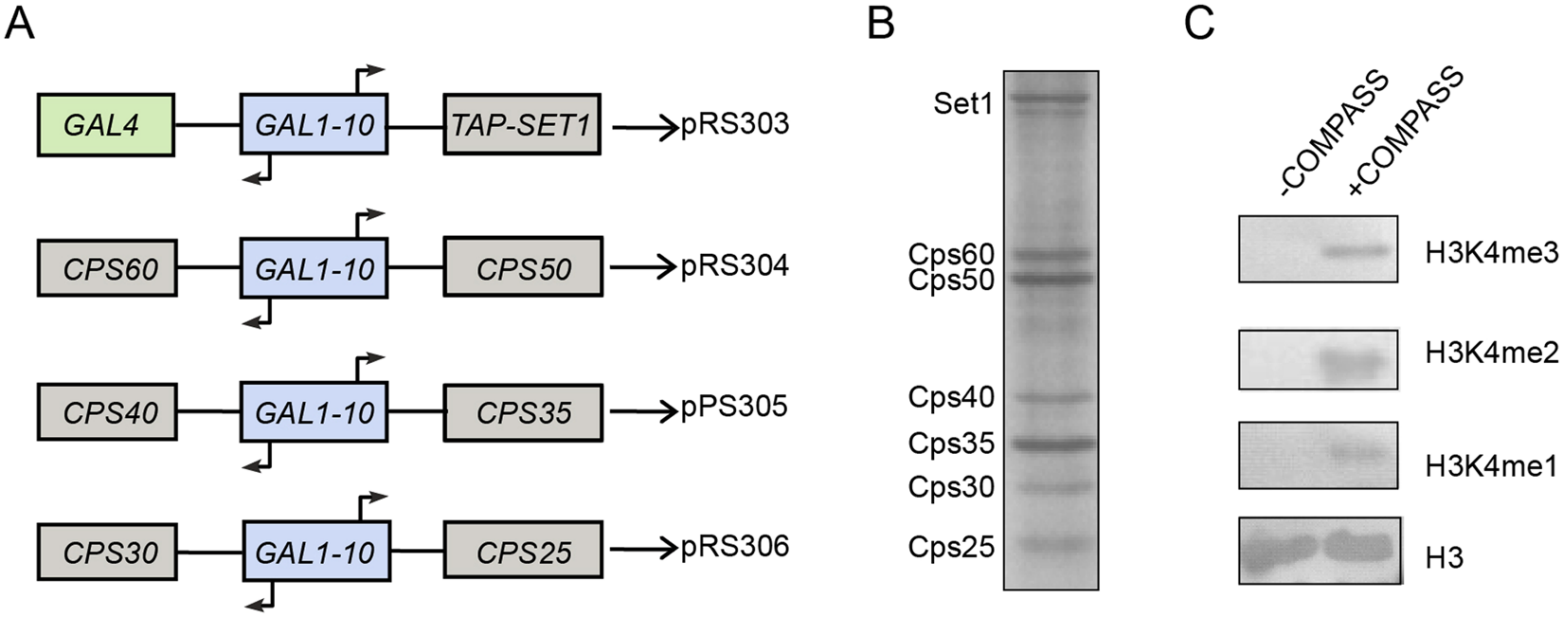
Overexpression and purification of the COMPASS complex from budding yeast *S*. *cerevisiae*. **(A)** COMPASS overexpression scheme. Full length *SET1* and the Cps subunits of COMPASS (*CPS60*, *CPS50*, *CPS40*, *CPS35*, *CPS30*, and *CPS25*) together with *GAL4* and *GAL1-10* were cloned into pRS30-series vectors. A TAP tag was fused to the N-terminus of Set1. **(B)** SDS-PAGE analysis of the purified COMPASS complex. Purified Set1 consistently runs on SDS/PAGE as a doublet, the reasons behind this property of Set1 or its biological significance remain unclear^7^. **(C)** *In vitro* H3K4 mono-, di-, and tri-methylation activities of COMPASS complex, examined by western blotting using H3K4me1, me2, and me3 antibody and anti-H3 antibody.

To obtain a sufficient quantity of COMPASS for cryo-EM study, we developed a strategy for overexpressing *S*. *cerevisiae* COMPASS in yeast. Four plasmids containing all seven subunits were generated making use of the bidirectional inducible *GAL1-10* promoter and the pRS30-series vectors (Fig. 1A). The subunits of COMPASS and *GAL4* genes were cloned and overexpressed in budding yeast. Here, GAL4 is a positive regulator of *GAL* genes in response to galactose, and overexpressed GAL4 could increase protein yield^30^. The complex was affinity-purified through TAP-tagged Set1, followed by glycerol gradient centrifugation. The resulting COMPASS complex consists of all seven components of the complex (Fig. 1B), and *in vitro* H3K4 methyltransferase activity assay validated its histone H3K4 methylation activity, confirming the conserved enzymatic activity in our overexpressed COMPASS complex (Fig. 1C). To further improve the integrity of the complex, we carried out GraFix after affinity purification with added crosslinker (glutaraldehyde)^31,32^, which indeed enhanced the integrity of the complex (Fig. S1).

### Architecture of the COMPASS complex revealed by cryo-EM

**Figure 2:**
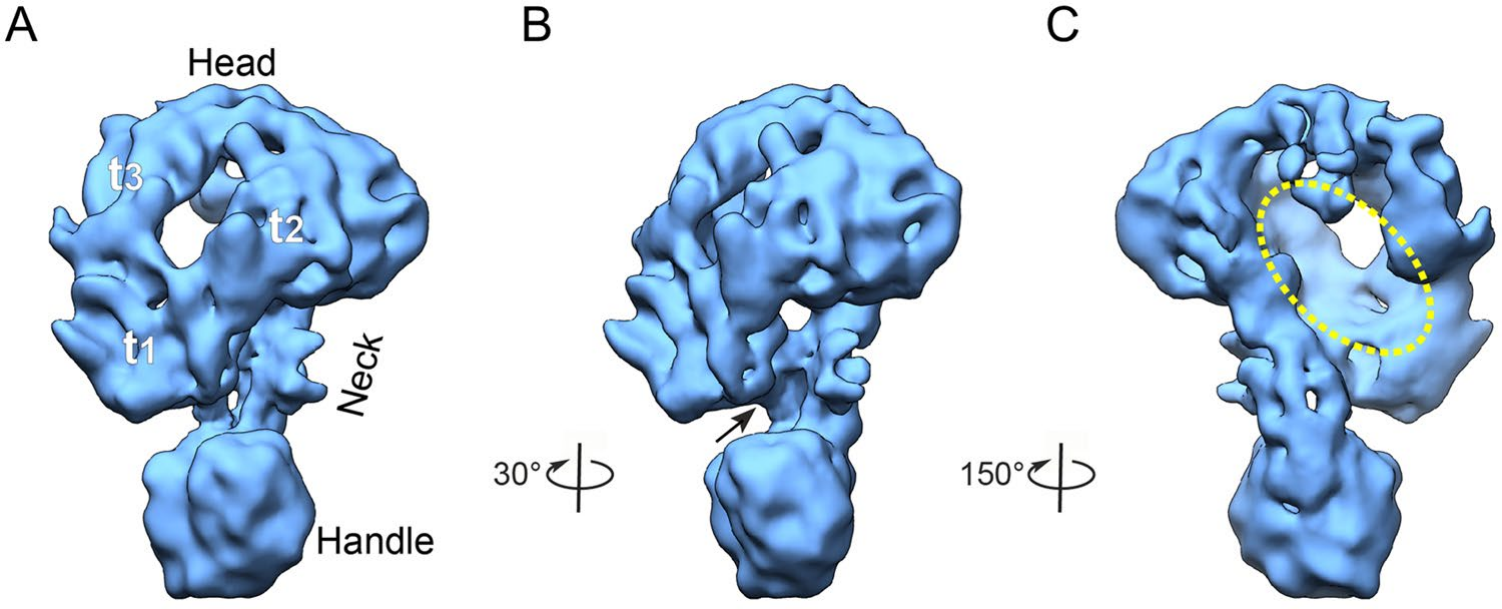
The architecture of the complete *S*. *cerevisiae* COMPASS complex. **(A)** Front view of the cryo-EM 3D map of COMPASS. Three portions of the head are labeled as t_1_, t_2_, and t_3_. The COMPASS complex is composed of three regions: head, neck, and handle. **(B, C)** Other representative views of the reconstructions, revealing the presence of (**B**) a groove formed between the head and the neck (indicated by black arrow), and (**C**) a canyon located in the back of the head (yellow ellipsoid).

The yeast COMPASS was imaged under a cryogenic condition, yielding 3,374 movies on a Titan Krios transmission electron microscope equipped with a K2 Summit direct electron detector (Fig. S2A,B and Table S1). A total of 364,188 particles were subjected to three rounds of 3D classifications. Eventually, three classes with 77,865 particles showing better structural details were further refined to a resolution of 10.0 Å (Figs 2, and S2C–E and S3). To our knowledge, this is the first complete structure of yeast COMPASS. Still, despite the relative high quality of the original movies (containing information up to ~4.0 Å resolution for most of the data, Fig. S2B) and a reasonable amount of particles (77,865) included in the final reconstruction, the resolution of the map was found to be relatively low. Our 2D and 3D analyses further prove that COMPASS is intrinsically dynamic (Movie S1) and compositionally heterogeneous (Fig. S3, 2^nd^ round classification). On the other hand, these dynamic natures might be beneficial for the involvement of different modules of the complex in distinct functions or biological activities^5,6,33^.

Our cryo-EM map reveals the architecture of COMPASS that can be divided into head, neck, and handle regions (Fig. 2). The head was observed to be ~110 Å in length, ~100 Å in height, and ~90 Å in width. Three portions of the head (labeled as t_1_, t_2_, and t_3_ in Fig. 2A) form a face-shaped structure perhaps corresponding to the three WD40 β-propeller-containing subunits including Cps50, Cps35, and Cps30. The neck connecting the head and the handle was measured to be ~40 Å in length. The handle located at the base of the reconstruction was observed to be ~55 Å in length, ~50 Å in width, and ~47 Å in height, and appears expanded than the neck. Interestingly, our map also showed a canyon (~90 Å in length, ~25 Å in width, and ~35 Å in depth) formed in the back of the head (Fig. 2C), and a groove formed between the head and the neck (Fig. 2B).

### Identification of key COMPASS subunits by using tag or Fab labeling strategy

**Figure 3:**
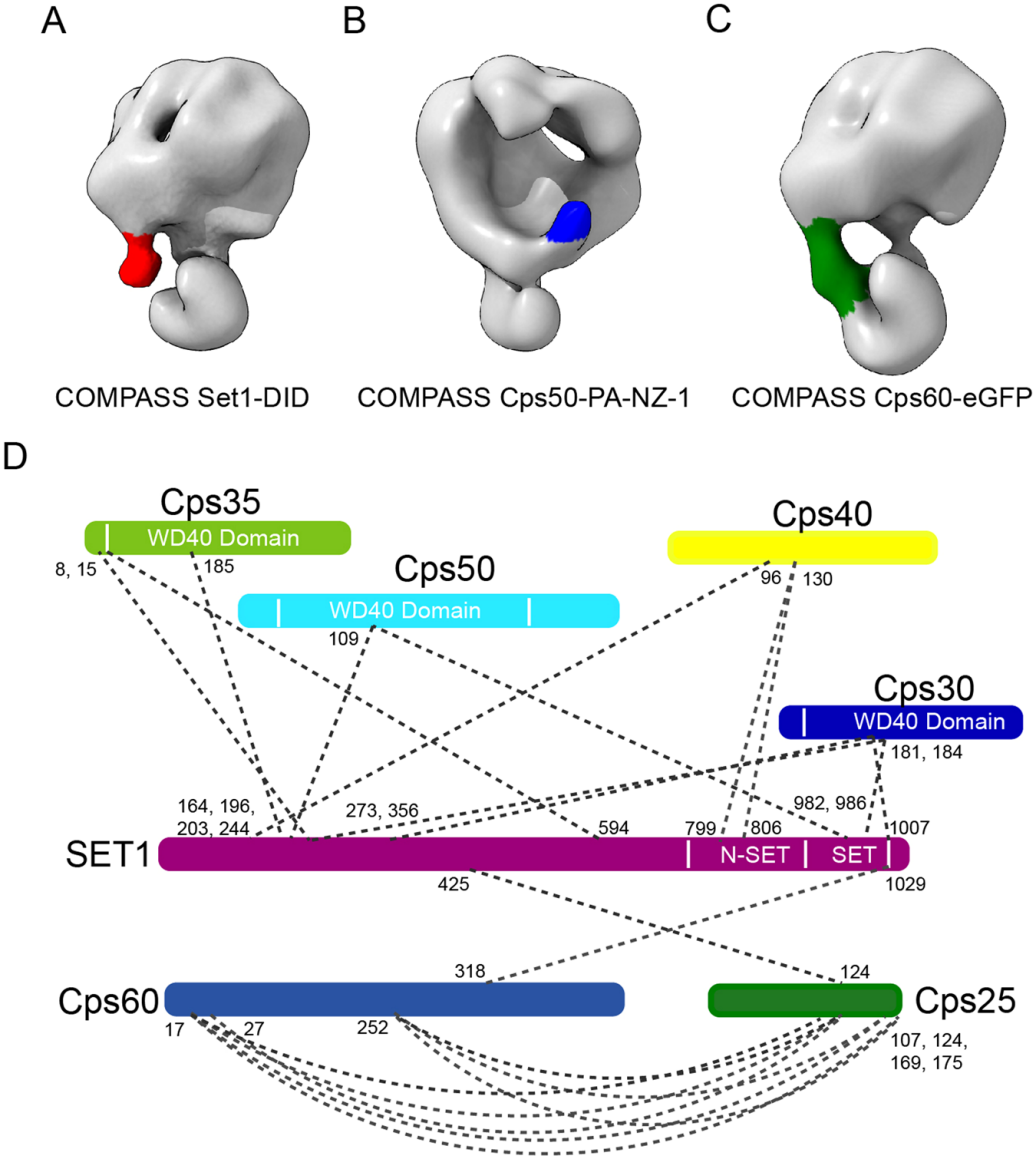
Key subunit mapping and XL-MS analysis on COMPASS complex. **(A**–**C)** Identification of the key COMPASS subunits determined through a tag or Fab labeling strategy. 3D cryo-EM maps of COMPASS Set1–DID (A), COMPASS Cps50–PA–NZ-1 (**B**), and COMPASS Cps60–eGFP (**C**), with COMPASS Set1–DID and COMPASS Cps60–eGFP showing the front view of the structure, and COMPASS Cps50–PA–NZ-1 showing the back of COMPASS. The density attributed to COMPASS is shown in grey, and that of DID, NZ-1 Fab, and eGFP, in red, green, and blue, respectively. **(D)** XL-MS analysis on COMPASS complex. Identified cross-linked contacts between different subunits are shown as dotted lines. We used Best e-value (1.00E-01) as the threshold to remove extra XL-MS data with lower confidence.

**Figure 4:**
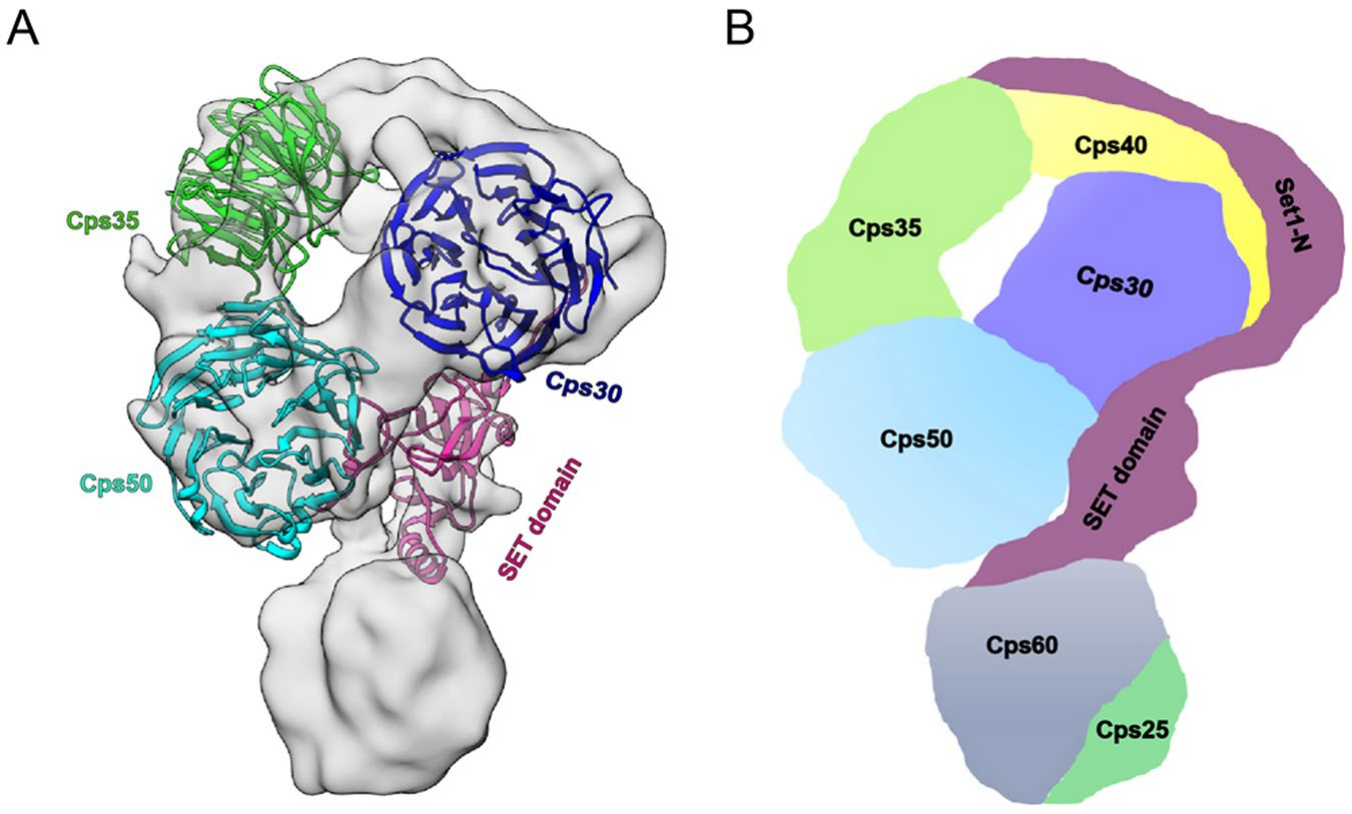
Subunit organization of yeast COMPASS. **(A)** Matches between the model and the cryo-EM map of COMPASS complex. (**B)** A cartoon diagram illustrating the potential subunit organization of COMPASS.

To decipher the exact locations of key subunits within the map, we used eGFP tag^24,25^, DID tag^26^, and our recently developed PA tag and NZ-1 Fab labeling strategies^27^. Set1 is the methyltransferase and the largest subunit of COMPASS^3,4^. To locate Set1 in the map, we fused DID tag^26^ (containing two diffident DID strands (DID1 and DID2) and six Dyn2 homodimers) to the C-terminus of Set1. The cryo-EM map of COMPASS Set1-DID shows an exposed piece of extra density at the jaw of the head and near the neck. This extra density is likely due to the DID tag (Figs 3A and S4A), suggesting the labeled Set1 C-terminus might locate near that spot. Besides, the SET domain of Set1 located in the C-terminal portion of Set1 has been reported to be situated at the junction of Cps50–Cps30 and Cps60–Cps25 modules^7^. Taken together, we located the SET domain in the neck of COMPASS connecting the head and handle where Cps50–Cps30 and Cps60–Cps25 locate, respectively, with its C-terminus expanding from the neck towards the jaw of the head (Fig. 4).

Cps50 and Cps30 can form a heterodimer in the absence of Set1^3^. Here, to locate Cps50-Cps30 heterodimer in the map, we adopted our recently developed yeast inner-subunit PA–NZ-1 epitope labeling strategy to insert a dodecapeptide PA tag^27–29^ into an exposed turn region in the WD40 domain of Cps50. The resulting cryo-EM map of COMPASS Cps50–PA–NZ-1 reveals an extra piece of density in the lower part of the head visualized from the back of the structure (Figs 3B and S4B), and this extra density was assigned to the NZ-1 Fab bound to the inserted PA tag. Therefore, our labeling experiment suggests Cps50 to be located in the lower part of the head, forming the t_1_ region (Figs 2A and 4). Moreover, regarding the location of Cps30, previous studies suggested Cps30 to be adjacent to both Cps50 and SET domain of Set1^7^, and human WDR5 (Cps30 homolog) directly interacts with the “*Win*” motif of MLL1^34^. We therefore assigned Cps30 to the t_2_ region in the head, which connects with both SET domain of Set1 in the neck and Cps50 (t_1_) in the head (Fig. 4).

To locate the Cps60-Cps25 heterodimer in the map, an eGFP tag was fused to the N-terminus of Cps60. This COMPASS Cps60-eGFP map yields an extra cylindrical piece of density bridging the handle and the head (Figs 3C and S4C), and this density is likely resulted from the eGFP tag. Having already assigned the related head density to Cps50, we then assigned Cps60 to the handle. Based on the labeling results, we specifically assigned Cps60 to the portion of the handle in close proximity to the head, while Cps25, the heterodimer partner of Cps60, could locate in the other side of the handle (Fig. 4B). This arrangement is in line with the previous report^7^, but with a more precise location assigned for Cps60. Note that in this eGFP-labeled map (Fig. 3C), the eGFP tag is prone to form a cylindrical bridge connecting the target subunit with an adjacent structural element in a suitable distance without knowing the reason. Still, combined with other structural information, we were allowed to assign the target Cps60 subunit.

### Homology model building and subunit assignment of COMPASS

To obtain a thorough picture of the subunit arrangement in the complex, we built homology models of COMPASS subunits (including Cps30, Cps35, Cps50, and SET domain of Set1) based on the *S*. *cerevisiae* sequences and available crystal structures of homologous proteins or domains as templates, whose sequence identities with the corresponding target subunits are higher than 25% to ensure producing relative reliable homology models^35,36^ (Table S2). We then manually docked the available models into the COMPASS cryo-EM map based on our subunit labeling results. Subsequently, to further improve the fitting, we used the simultaneous multi-fragment refinement program *collage* from Situs software package^37^ to refine the manually fitted multi-fragment model of the COMPASS complex (Fig. 4A).

The SET domain of Set1 was docked into the neck of the reconstruction with the C-terminus of SET domain extending towards the jaw of the head (Fig. 4A), and its N-terminus pointing to the top of the head. We then proposed the remaining N-terminal portion of Set1 to stretch out from the neck to the top of the head. Also, having already assigned the WD40 domain-containing subunits Cps50 and Cps30 to t_1_ and t_2_ regions of the head, respectively, the remaining WD40-containing subunit Cps35 was then docked into the remaining t_3_ position of the head (Fig. 4A). Overall, the models of the three WD40-containing subunits were found to match the density in the face region well (Fig. 4A). Still, there is remaining unoccupied density at the top of the head. A previous study suggested that Cps40 interacts with the n-SET domain of Set1, and sits tightly on the top of the Cps50–Cps30 module^9^. We thus postulated Cps40 to be located at the top of the head, and the unoccupied density in this region to belong to Cps40 and the un-modeled portion of Set1 (Fig. 4). Cps60 was assigned into the portion of the handle close to the head (Fig. 4A). In this way, we proposed the positions of all the subunits in our cryo-EM map of the yeast COMPASS complex, and their arrangement is in agreement with available structural and biochemical information^7,9,21^.

### Dissecting subunit interactions by cross-linking and mass spectrometry

To characterize the subunit interaction network and validate the subunit assignment of COMPASS, we performed additional XL-MS analysis on the purified COMPASS complex (Table S3). This XL-MS data revealed that all the other six subunits were cross-linked with Set1. This XL-MS data combined with our structural analyses indicated the critical role played by Set1 in maintaining the integrity of the full complex. Cps30, Cps50, and Cps60 were cross-linked to the SET domain of Set1 (Fig. 3D). Cps30 and Cps50 were also found to cross-link with the N-terminal region of Set1. Cps40 was found to cross-link to the n-SET domain of Set1, consistent with previous reports^9,38^. Previous study revealed direct binding of Cps35 to the N-terminal portion (1–229) of Set1^38^, our XL-MS data also suggest that Cps35 is cross-linked to this portion of Set1. Furthermore, XL-MS analysis revealed that Cps60 and Cps25 are extensively cross-linked to each other, with the C-terminal region of Cps25 cross-linked to the N-terminal region of Cps60. Putting together, the subunit interaction network provided by the XL-MS analysis substantiates our subunit assignment through the tag or Fab labeling experiments and the model docking results (Figs 3A–C and 4).

## Discussion

COMPASS is a multi-protein assembly playing an essential role in histone H3K4 methylation. In this study, we overexpressed all the seven full-length yeast COMPASS subunits, which formed the complete complex showing histone H3K4 methyltransferase activity (Fig. 1). Our cryo-EM map revealed the architecture of the complete COMPASS complex (Fig. 2). Based on a combination of tag or Fab labeling strategy (including DID, eGFP, and PA–NZ-1) with cryo-EM 3D reconstruction (Figs 3A–C and S4), XL-MS analysis (Fig. 3D), model fitting, and available subunit interaction information, we assigned all of the subunits to specified portions of the COMPASS cryo-EM map (Fig. 4). Our study produced the first snapshot of the full molecular architecture of yeast COMPASS complex and a picture of its subunit interaction network.

Our results showed that the three WD40 domain-containing subunits of the COMPASS locate in the head of the complex, specifically in a face-shaped structure, with Cps50 in the lower t_1_ position, Cps30 in the upper t_2_ position connecting with Cps50 and the neck of the complex, and Cps35 in the upper t_3_ position adjacent to Cps50 (Fig. 4). The data suggested SET domain of Set1, the catalytic center of COMPASS, to be located in the neck, with the N-terminal portion of Set1 extending to the top of the head and interacting with Cps30 and Cps35 (Fig. 4), consistent with our XL-MS results. Cps60 and Cps25 are adjacent to each other and locate in a handle-shaped region of the complex, with Cps60 close to the head (Fig. 4B).

Our study provided new insights into the subunit interaction network of yeast COMPASS. The SET domain locates in the neck connecting the head and the handle, interacting with Cps60 and Cps50, respectively, forming the H3K4 methylation active center of the complex (Fig. 4). This arrangement is in general in line with a previous report showing the MLL SET domain on its own to adopt an open conformation, and to be induced into a closed conformation when interacting with RbBP5 and ASH2L (Cps50 and Cps60 homologs, respectively)^18,21^. Interestingly, this active center was observed in our reconstruction to be situated at the groove formed between the head and the neck and to be also adjacent to the handle (Fig. 4). The dynamic SET domain, located in the neck, may thus endow the active center with increased plasticity, beneficial for the complex to perform its H3K4 methyltransferase function. Furthermore, the N-terminal region of Set1, located at the top of the head, may act as a scaffold to stabilize the interactions between the regulatory subunits (Fig. 4B).

The WDR5 (Cps30 homolog) binding site on MLL1 (Set1 homolog) has been mapped to the conserved “*Win*” motif^19,34^. In our model, Cps30 interacts with Set1 in a region apart from the catalytic SET domain of Set1, with this region perhaps corresponding to the “*Win”* motif of Set1. We additionally showed Cps30 to interact with both Set1 and Cps50, consistent with previous reports^3,7,17^. Therefore, Cps30 may function as a binding platform to bridge Set1 and Cps50, and hence may facilitate their allosteric cooperativity and enhance the catalytic activity of Set1.

We assigned Cps35 to the upper portion of the head, in close proximity to Cps40 and the N-terminal region of Set1 (Fig. 4), together forming an upper peripheral region of the complex. A network of interactions between these components was also indicated by our XL-MS results, which showed that Cps35 was cross-linked to the N-terminal region of Set1, and Cps40 was cross-linked with the n-SET domain of Set1 (Fig. 3D). Consistently, a previous biochemical study suggested that Cps35 binds directly to the N-terminal extension (1–229) of Set1, and Cps40 binds to the n-SET domain of Set1^38^. Among these peripheral subunits, Cps40 was reported to bind H3K4me3 and interact with a double-strand break (DSB) protein, Mer2, to promote DSB formation close to gene promoters^39^. Cps35 in conjunction with the monoubiquitination machinery (Rad6/Bre1 and the interacting factors) functions to focus the H3K4me3 activity of COMPASS at the promoter-proximal regions of the gene^9^. Another study indicated that Set1A/Set1B COMPASS is recruited to chromatin by Wdr82 (Cps35 homolog)^40,41^. In hematopoietic progenitor cells, Nup98 interacts with Wdr82 to recruit the Set1A/COMPASS complex to promoters so as to regulate H3K4 trimethylation^42^. Therefore, the upper peripheral location of Cps35, Cps40, and the N-terminal region of Set1 uncovered in this study could be beneficial for their interaction with other cofactors or machineries involved in crosstalk with other functions.

It has been reported that Cps60 and Cps25 can form heterodimer in the absence of Set1. Our XL-MS data also showed that Cps60 and Cps25 are extensively cross-linked to each other through the C-terminal region of Cps25 and the N-terminal region of Cps60. Our study additionally suggested that Cps60, located in the handle with Cps25, interacts with the catalytic SET domain to participate in the regulation of COMPASS holoenzyme activity together with Cps50 and other subunits. Interestingly, we observed a canyon (formed by Set1, Cps50, Cps30, and Cps35) in the back of the head (Fig. 2C), which might be a potential nucleosome core particle (NCP) binding site in yeast COMPASS.

In summary, we determined the architecture of the complete yeast COMPASS complex and outlined the subunit arrangement. Our map also showed the location of the active site of the complete COMPASS, and revealed a canyon in the back of the head that may be the binding site for NCP. This study provides new insights into the machinery of COMPASS. Moreover, our studies on yeast COMPASS may pave the way towards understanding the role of mammalian COMPASS family in the regulation of H3K4 methylation.

## Materials and Methods

### Plasmids and yeast strains

The plasmids and strains used in this study are listed in Table S4 and Table S5, respectively. The COMPASS complex was overexpressed in yeast strain yCOS1. Full length *SET1* and the Cps subunits of COMPASS (including *CPS60*, *CPS50*, *CPS40*, *CPS35*, *CPS30*, *and CPS25*) together with *GAL4* and *GAL1-10* were cloned into pRS30-series vectors. The N-terminus of Set1 was fused with a TAP tag, and *GAL4*, *GAL1-10*, and *TAP-SET1* were cloned into pRS303 (to form IV1). *CPS60*, *GAL1-10*, and *CPS50* were cloned into pRS304 (to form IV2); *CPS40*, *GAL1-10*, and *CPS35* into pRS305 (to form IV3); and *CPS30*, *GAL1-10*, and *CPS25* into pRS306 (to form IV4) (Fig. 1A). These four vectors were integrated into the strain of yCOS1, yielding yCOS2.

To overexpress the DID tag, *DID1* was cloned into IV1 in the C-terminus of *SET1* (to form IV1-D). To avoid *in vivo* dimerization, the intrinsic Dyn2 of yCOS1 was knocked out (to form yCOS3). IV1-D, IV2, IV3, and IV4 were integrated into the strain of yCOS3 (to form yCOS4). Yeast *dyn2* and *DID2* were amplified and cloned into pET28a-GSTTEV and pET28a-HISTEV, respectively. These vectors were transformed into BL21 (DE3) respectively.

To overexpress COMPASS with PA-labeled Cps50 or eGFP-labeled Cps60, the endogenous *CPS60* or *CPS50* of yCOS1 was knocked out, producing the yCOS5 or yCOS6 strains, respectively. The *eGFP* gene was inserted into IV2 at the N-terminus of *CPS60* (to form IV2-60E). IV2-60E together with IV1, IV3, and IV4 were integrated into yCOS5 (to form yCOS9). A Cps50-PA construct was prepared by inserting the PA tag^29^ into IV2, between I134 and F135 of Cps50. The PA tag gene was inserted into *CPS50* using site-directed mutagenesis, yielding IV2-50PA. IV2-50PA together with IV1, IV3, and IV4 were integrated into yCOS6 (to form yCOS11).

### Protein preparation

To overexpress COMPASS and eGFP- or PA-labeled COMPASS, ten liters of the cells were grown in YP-raffinose at 30 °C to an optical density, specifically OD_600nm_ of 0.8-1.0, and protein expression was induced by adding galactose (2%) to the grown cells and incubating the resulting mixture at 30 °C for 3–4 h. Cells were collected by centrifuging the incubated mixture at 5000 g for 10 min. The collected cells were resuspended in lysis buffer (50 mM Tris-HCl [pH 8.0], 150 mM NaCl, 10% glycerol, 0.1% NP-40, 1 mM EDTA, 1 mM DTT, protease inhibitors (Roche)) and then disrupted by using a high-pressure homogenizer. The resulting mixture including disrupted cells was subjected to centrifugation at 25,000 g for 30 min, and the supernatant was incubated with IgG-Sepharose beads (GE Healthcare) for 3 h at 4 °C. These beads were recovered, washed with wash buffer (50 mM Tris-HCl [pH 8.0], 350 mM NaCl, 10% glycerol, 0.05% NP-40, 1 mM DTT) and TEV cleavage buffer (50 mM Tris-HCl [pH 8.0], 150 mM NaCl, 10% glycerol, 0.05% NP-40, 0.5 mM EDTA, 1 mM DTT), and then incubated with TEV protease overnight at 4 °C. The eluate of this process was recovered, followed by being combined with 5 mM CaCl_2_ and then incubated with calmodulin-Sepharose (GE Healthcare) at 4 °C for 3 h. This incubated eluate was washed with CBP wash buffer (50 mM Tris-HCl [pH 8.0], 150 mM NaCl, 10% glycerol, 2 mM CaCl_2_, 0.05% NP-40, 1 mM DTT). COMPASS and eGFP/PA-labeled COMPASS were each eluted using CBP elution buffer (50 mM Tris-HCl [pH 8.0], 150 mM NaCl, 10% glycerol, 2 mM EGTA, 0.05% NP-40, 1 mM DTT).

The eluate in each case was concentrated and further purified using glycerol gradient centrifugation or GraFix^31,32^. Each protein sample was applied to a 10–40% (w/v) glycerol gradient with/without a 0–0.1% glutaraldehyde gradient in the GraFix buffer (50 mM HEPES [pH 8.0], 150 mM NaCl, 1 mM DTT). The resulting gradients were subjected to ultracentrifugation at 4 °C for 16 h at 41000 rpm in an SW41 Ti rotor (Beckman), and then fractionated. Residual glutaraldehyde in the fractions was neutralized by adding Tris-HCl (pH 8.0) to a final concentration of 50 mM. The fractions were concentrated for biochemical and cryo-EM analyses.

Dyn2 and DID2 were expressed in BL21 (DE3). Two liters of the cells were grown in Luria-Bertani medium, at 37 °C, to an OD_600nm_ of 0.6–0.8. IPTG at a final concentration of 0.5 mM was then added to the cell suspension. Induction was carried out overnight at 16 °C. The Flag-DID2 and Dyn2 proteins were purified as previously described^26^. Furthermore, to prepare DID-labeled COMPASS, excess amounts of purified Dyn2 and Flag-DID2 were incubated with calmodulin-Sepharose beads carrying the immobilized COMPASS complex with Set1-DID1 overnight at 4 °C. The COMPASS labeled with the DID tag was eluted and further purified using GraFix.

### *In vitro* H3K4 methyltransferase analysis

Recombinant COMPASS complexes (without glutaraldehyde) were incubated with 0.5 μg of free histone H3 and 250 μM S-adenosylmethionine in methyltransferase reaction buffer (50 mM HEPES [pH 8.0], 150 mM NaCl, 1 mM DTT) for 2 h at 30 °C. The extend of methylation of histone H3 was examined using Western analysis with anti-H3K4me1 (Abcam, ab8895), anti-H3K4me2 (Abcam, ab7766), anti-H3K4me3 (Abcam, ab8580), and anti-H3 (Abcam, ab18521) antibodies.

### Analysis of the purified complex by negative staining EM

5 μl of the sample after GraFix was deposited onto a glow-discharged 400 mesh continuous carbon grid (Beijing Zhongjingkeyi Technology Co., Ltd.) and stained with 2% uranyl acetate. Data were collected by utilizing a Tecnai T12 transmission electron microscope (FEI company, USA) operated at an acceleration voltage of 120 kV. Micrographs were acquired at a nominal microscope magnification of 67,000 using a 4k x 4k Eagle CCD camera. Images were collected at a defocus ranging from −1 to −2 μm with a pixel size of 1.74 Å/pixel.

### Cryo-EM sample preparation and data collection

For cryo-EM sample preparation, a volume of 2 μl of the cross-linked COMPASS sample was placed onto a holey carbon grid (Quantifoil R1.2/1.3, 200 mesh), which was blotted with a Vitrobot Mark IV (FEI) and then plunged into liquid ethane cooled by liquid nitrogen. For PA tag inserted COMPASS, the sample was first incubated with NZ-1 Fab^29^ on ice for 30 min, and then prepared for vitrification as described.

Images were acquired on a Titan Krios transmission electron microscope (FEI) operated at 300 kV and equipped with a Cs corrector. The images were recorded on a K2 Summit direct electron detector (Gatan) in counting mode with a pixel size of 1.32 Å/pixel. Each movie was dose-fractioned into 38 frames, with a total accumulated dose of ~47 e^−^/Å^2^ on the specimen (Table S1). All of the images were collected by utilizing SerialEM, the automated data collection software package^43^ with the final defocus values varied from −0.9 to −3.0 μm.

### Image processing and 3D reconstruction

A total of 3,374 micrographs were used for COMPASS structure determination (Supplementary information, Table S1). All images were aligned and summed using MotionCorr whole-image motion correction software^44^ (Fig. S2A,B). Unless otherwise specified, single-particle analysis was mainly performed using RELION 1.3^45^ (Fig. S3). After CTF parameter determination using CTFFIND3^46^, particle auto-picking, manual particle checking, and reference-free 2D classification, 364,188 particles were yielded for further processing. Initial model building was carried out using EMAN1.9 software package^47^: based on the 2D class-averages of 10,978 particles (using *refine2d*.*py* program), we performed initial model building by utilizing *startAny* program, which was refined using *refine* program. We then used this yielded map as the initial model (Fig. S3). One round of 3D classification over the entire dataset was carried out to generate four classes, which allowed us to extract 199,502 particles in one class (class 1) with better structural features. These particles went through an auto-refine procedure with a soft mask to generate a map. We consider this is a more reliable model to classify the particles. We then used this map as the input model to perform another round of 3D classification over the entire dataset, which allowed us to obtain a class with more complete and detailed structural features (class 4) containing 95,529 particles. Further refinement on this class gave a map showing better structural details. Moreover, we used this map as the input model to perform another round of 3D classification over the 95,529 particles. After excluding one class with bad structural features, we further refined the remaining 77,865 particles and obtained a map at ~10.0 Å resolution. The resolution was accessed based on the gold-standard criterion with FSC at 0.143, and the local resolution was estimated by ResMap^48^.

For the tag/Fab-labeled COMPASS datasets, we used the unlabeled COMPASS map as the initial model but low-pass filtered to 60 Å, and the aforementioned reconstruction procedure was followed.

### Homology model building

For homology model building of the individual subunits of yeast COMPASS, we took the corresponding *S*. *cerevisiae* sequences from the Saccharomyces Genome Database (http://www.yeastgenome.org/), and used the SWISS-MODEL webserver for model building^49–51^ (https://swissmodel.expasy.org/). Due to the limited available structural information on homologous templates, we built the homology models of SET domain of Set1 (916–1080), Cps50 (20–341), Cps35 (30–328), Cps30 (8–314), whose sequence identities with the templates are higher than 25% to ensure a relative reliable homology model building (Table S2).

We first manually fitted the related homology models into the corresponding positions in the map as rigid bodies based on our subunit mapping results from labeling experiments and other structural analyses. We then used the simultaneous multi-fragment refinement program *collage* from Situs to further refine the multi-fragment model against the map^37^. All of the figures were rendered by utilizing UCSF Chimera and ChimeraX^52^.

### Cross-linking and mass spectrometry analysis

The purified yeast COMPASS from glycerol gradient was cross-linked by disuccinimidyl suberate (DSS), with a final concentration of crosslinker at 1 mM. 20 mM Tris-HCl was used to terminate the reaction after incubation on ice for 2 hours. Cross-linked complexes were precipitated with cooled acetone and lyophilized. The pellet was dissolved in 8 M urea, 100 mM Tris pH 8.5, followed by TCEP reduction, iodoacetamide alkylation, and overnight trypsin (Promega) digestion. Digestion was quenched by 5% formic acid. Tryptic peptides were desalted with MonoSpin C18 spin column (GL Science) and then separated within a home packed C18 column (Aqua 3 μm, 75 μm × 15 cm, Phenomenex) in a Thermo EASY-nLC1200 liquid chromatography system by applying a 60-minute step-wise gradient of 5–100% buffer B (84% acetonitrile (ACN) in 0.1% formic acid). Peptides eluted from the LC column were directly electrosprayed into the mass spectrometer with a distal 2 kV spray voltage. Data-dependent tandem mass spectrometry (MS/MS) analysis was performed with a Q Exactive mass spectrometer (Thermo Fisher, San Jose, CA). Raw data was processed with pLink software^53^ and Proteome Discoverer 2.2 xlinkx (Supplementary information, Table S3).

## Electronic supplementary material

supplementary information

## Data Availability

Cryo-EM map of yeast COMPASS complex has been deposited in the EMDB (EMD-9694). Other data that support the findings of the study are available from the corresponding author upon request.
