# Supplementary material for: Architecture and subunit arrangement of the complete Saccharomyces cerevisiae COMPASS complex
Source: Sci Rep. 2018 Nov 27;8:17405. doi: 10.1038/s41598-018-35609-8 (PMC6258710; doi:10.1038/s41598-018-35609-8)
Supplement: Supplementary file 1 — supplementary information [file 41598_2018_35609_MOESM1_ESM.pdf]

Architecture and subunit arrangement of the complete *Saccharomyces cerevisiae* COMPASS complex

Yanxing Wang[1,2,#], Zhanyu Ding[1,#], Xiangyang Liu[1], Yu Bao[1], Min Huang[1], Catherine C. L. Wong[3], Xiaoyu Hong[1], Yao Cong[1,2,*]

[1]National Center for Protein Science Shanghai, State Key Laboratory of Molecular Biology, CAS Center for Excellence in Molecular Cell Science, Shanghai Institute of Biochemistry and Cell Biology, Chinese Academy of Sciences, University of Chinese Academy of Sciences, Shanghai, China 201210

[2]Shanghai Science Research Center, Chinese Academy of Sciences, Shanghai, China 201210

[3]Center for Precision Medicine Multi-Omics Research, Peking University Health Science Center; State Key Laboratory of Natural and Biomimetic Drugs, School of Pharmaceutical Sciences, Peking University, Beijing,China 100191

[#]These two authors contributed equally to this work.

*Correspondence should be addressed to Y.C. (cong@sibcb.ac.cn).

Supplementary Figures

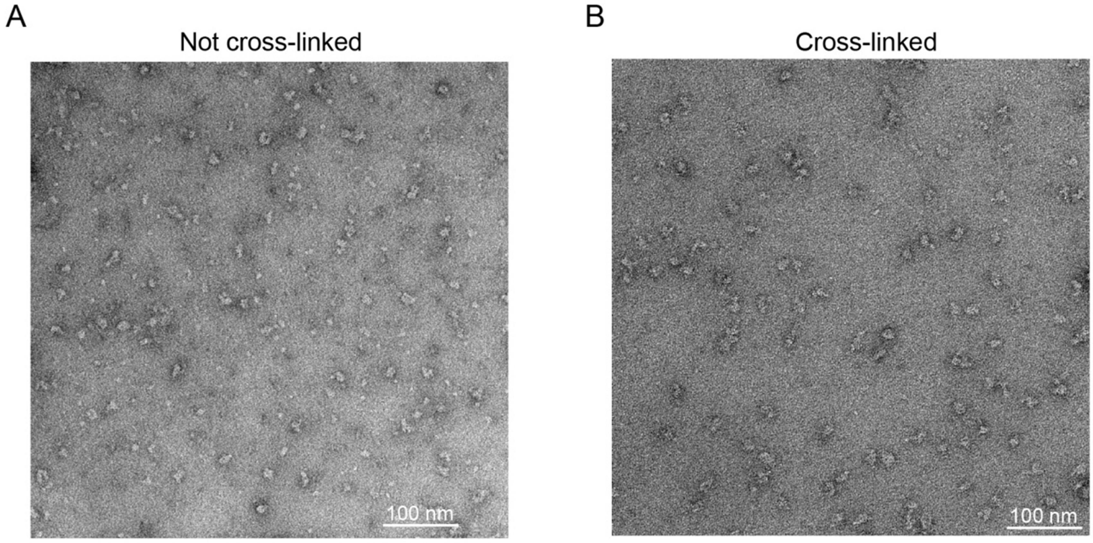

Figure S1. Integrity enhancement of the purified yeast COMPASS complex by cross-linking. (A) Representative negative-stain EM images of the COMPASS complex without cross-linking. (B) Representative negative-stain EM images of the complex after cross-linking by glutaraldehyde through GraFix procedure. Cross-linking indeed helps to enhance the integrity of the complex.

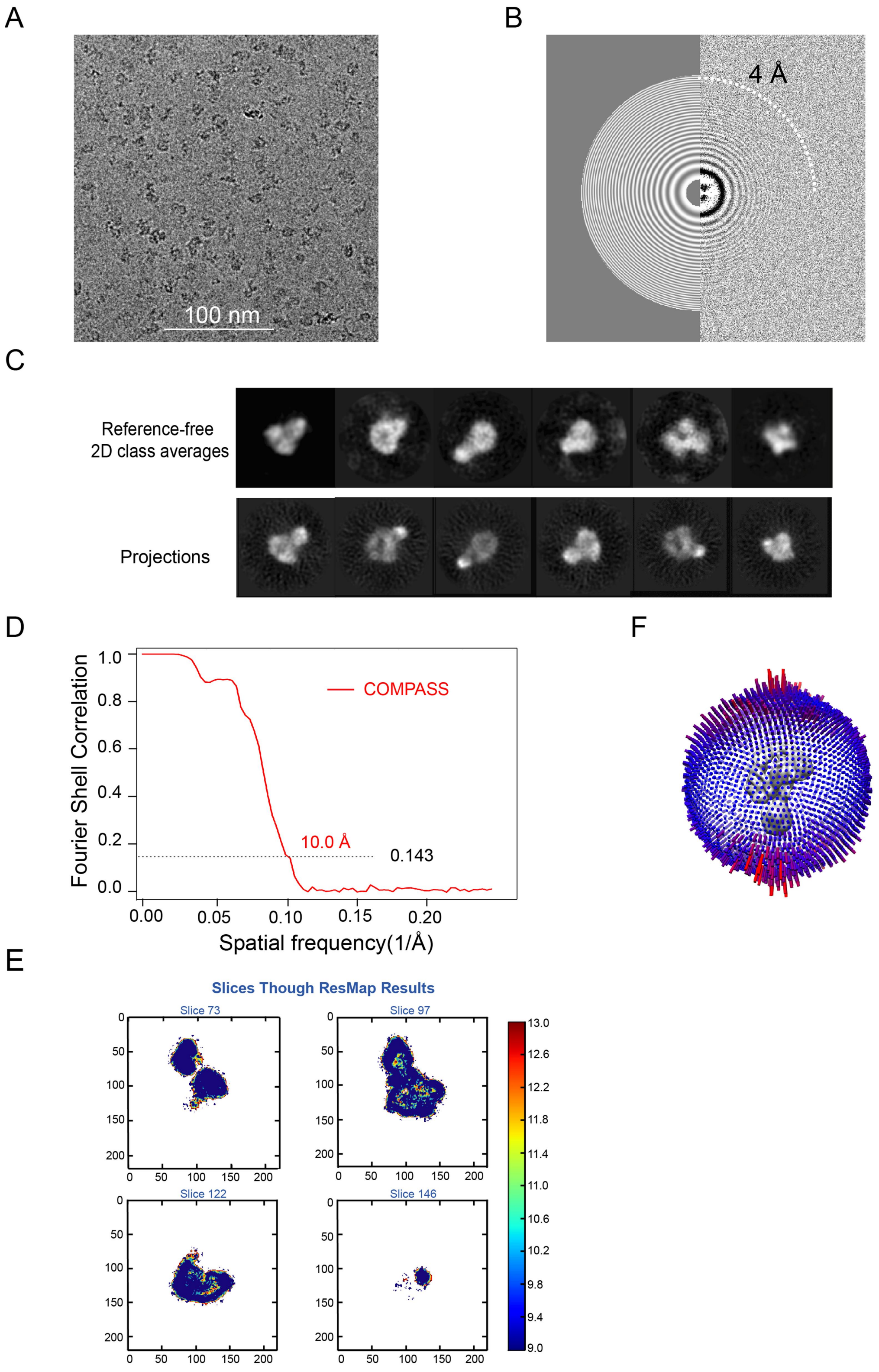

Figure S2. Cryo-EM analysis of the COMPASS complex. (A) Representative cryo-EM image of the COMPASS complex.For better visualization, the original data

were low-pass filtered to 10 Å to enhance the contrast. (B) The 2D power spectrum of the original macrograph, indicating the presence of information in the data up to a resolution of 4 Å. (C) Representative projections of the final cryo-EM map of the COMPASS complex (bottom row) and the corresponding reference-free 2D class averages (top row) obtained from raw particles showing similar structural features. (D) Resolution estimation of the cryo-EM map of the complete COMPASS complex according to the gold-standard FSC criterion of 0.143. (E) Local resolution estimation of the COMPASS map by Resmap. The color bar on the right labels the corresponding resolution (in Å). (F) The angular distribution of the 3D reconstructions, indicating unequal occupancy of angular classes.

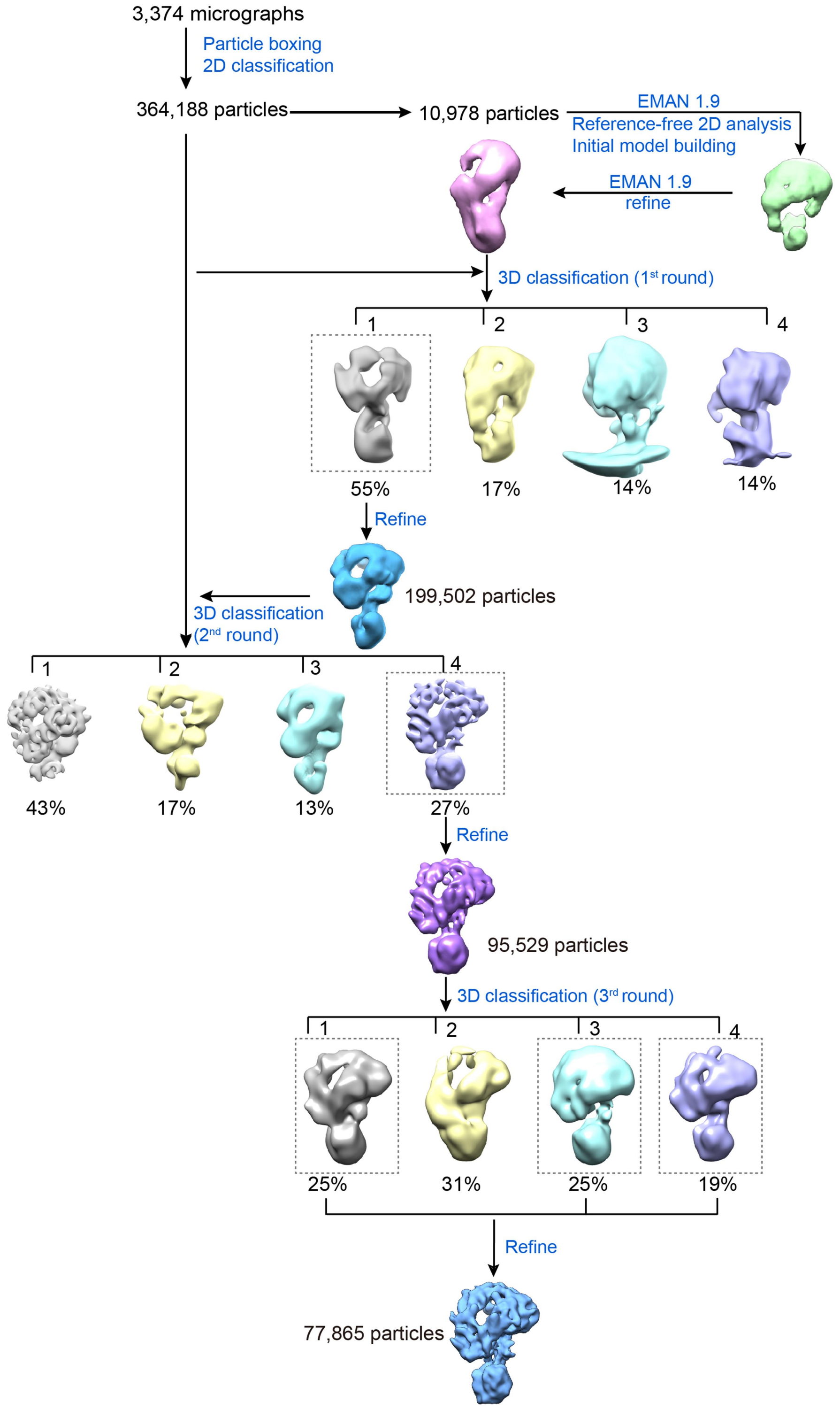

Figure S3. Work flow for the processing of cryo-EM data. After particle auto-picking, manual checking, and reference-free 2D classification, 364,188 particles

were yielded for further processing. The initial model was generated based on the 2D class-averages of 10,978 particles and further refined using EMAN1.9 software package. One round of 3D classification over the entire dataset was carried out to generate four classes, which allowed us to extract 199,502 particles in one class (class 1) with better structural features. These particles went through an auto-refine procedure with a soft mask to generate a map. We consider this is a more reliable model to classify the particles. We then used this map as the input model to perform another round of 3D classification over the entire dataset, which allowed us to obtain a class with more complete and detailed structural features (class 4) containing 95,529 particles. Further refinement on this class gave a map showing better structural details. Moreover, we used this map as the input model to perform another round of 3D classification over the 95,529 particles. After excluding one class with bad structural features, we further refined the remaining 77,865 particles and obtained a map at ~10.0 Å resolution.

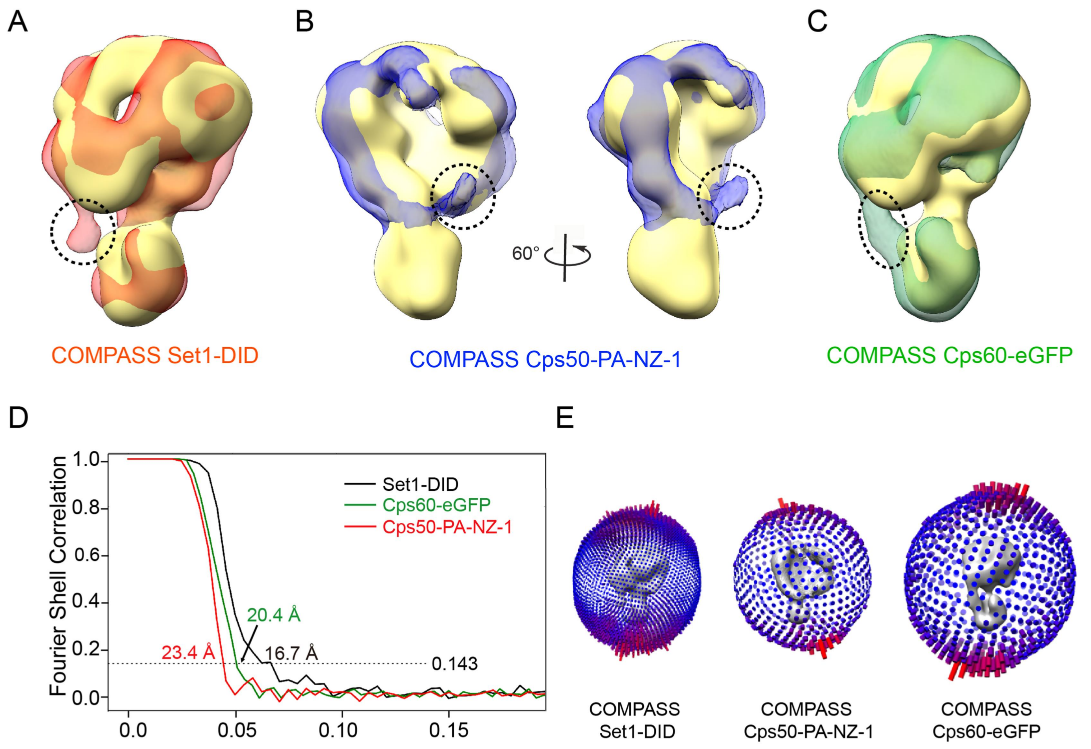

Figure S4. Identification of key COMPASS subunits determined through a tag or Fab labeling strategy. (A) Alignment of the COMPASS Set1–DID map (red transparent) with a blurred COMPASS map (in khaki surface throughout this figure). A patch of exposed extra density (indicated by black circle) is visible in the COMPASS Set1–DID map, compared with the COMPASS map. Showing is the front view. (B) Alignment of the COMPASS Cps50–PA–NZ-1 map (blue transparent) with the blurred COMPASS map. A patch of exposed extra density corresponding to the NZ-1 Fab is indicated by black circle. To better visualize the extra density, we showed both the back view and a rotated view. (C) Alignment of the COMPASS Cps60–eGFP map (green transparent) with the blurred COMPASS map. A patch of exposed extra density corresponding to eGFP is indicated by black ellipsoid. Showing is the front view. (D) Resolution estimation of thecryo-EM maps of the labeled COMPASSaccording to the gold-standard FSC criterion of 0.143. (E) The angular distribution of the 3D reconstructions, indicating unequal occupancy of angular classes.

Supplementary Tables

Table S1. Cryo-EM data collection and refinement statistics

	COMPASS	COMPASS Set1-DID	COMPASS Cps60-eGFP	COMPASS Cps50-PA-NZ-1
**Data collection**				
EM equipment	Titan Krios	Titan Krios	Titan Krios	Titan Krios
Voltage (kV)	300	300	300	300
Detector	K2 Summit	K2 Summit	K2 Summit	K2 Summit
Pixel size (Å)	1.32	1.32	1.32	1.32
Total electron dose ($e^-/Å^2$)	47	47	47	47
Dose rate (e^-/physical pixel/sec)	10	10	10	10
Exposure time (s)	7.6	7.6	7.6	7.6
Frames	38	38	38	38
Defocus range (µm)	-0.9 ~ -3.0	-1.0 ~ -3.0	-0.8 ~ -4.0	-1.5 ~ -3.2
**Reconstruction**				
Software	RELION 1.3	RELION 1.3	RELION 1.3	RELION 1.3
Raw micrographs	3,374	589	671	172
Original particles	364,188	144,612	81,432	19,163
Final particles	77,865	92,254	31,482	8,510
Symmetry	C1	C1	C1	C1
Final resolution (Å)	10.0	16.7	20.4	23.4

Table S2. Summary of homology model building for the yeast COMPASS

Model	Coverage	Template structure	Sequence identity
SET domain	916-1080	2W5Y	46.43%
Cps30	8-314	5SXM	38.41%
Cps35	30-328	3N0E	27.27%
Cps50	20-341	5OV3	30.08%

Table S3. Results of XL-MS analysis of yeast COMPASS complex

Protein1(site)-Protein2(site)	Peptides	Best e-value	Spec count
Cps60(27)-Cps25(124)	NALQGQQEGKRPNLPQMEATHQIK(10)-KYLNTNVTPHLLAGMR(1)	9.80E-13	1
Cps60(17)-Cps25(175)	LGIIPYQEGTDIVYKNALQGQQEGK(15)-SGEKESNASK(10)	1.08E-12	3
Cps25(169)-Cps60(252)	SGEKESNASK(4)-QAKEFTK(3)	9.03E-11	3
Cps40(130)-Set1(806)	LKTDEDR(2)-KVLEEEK(1)	2.36E-09	9
Cps60(17)-Cps25(124)	LGIIPYQEGTDIVYKNALQGQQEGK(15)-KYLNTNVTPHLLAGMR(1)	1.16E-08	5
Cps60(456)-Cps35(15)	IISEEDKLEYLDQIRSAYCVDGNSK(7)-FKHVKSFQPQEK(2)	4.89E-08	1
Set1(806)-Cps40(130)	RKVLEEEK(2)-LKTDEDR(2)	7.85E-08	10
Set1(521)-Cps15(2)*	SSSSSNSTNVPIKYESK(13)-AYNQEDSKR(1)	8.32E-08	12
Cps25(175)-Cps60(252)	SGEKESNASK(10)-QAKEFTK(3)	1.82E-07	7
Cps60(17)-Cps25(169)	LGIIPYQEGTDIVYKNALQGQQEGK(15)-SGEKESNASK(4)	3.65E-06	3
Cps60(318)-Set1(1029)	YKNQLFFEATDYVK(2)-IIKVGGR(3)	5.37E-06	10
Cps35(185)-Set1(196)	KIQEGPFLIIK(1)-IKHR(2)	1.23E-05	2
Cps25(175)-Cps60(256)	SGEKESNASK(10)-EFTKR(4)	1.27E-05	2
Cps30(181)-Set1(356)	TLTYDKDWK(6)-FVEINVKK(7)	7.62E-04	1
Cps25(124)-Cps60(252)	KYLNTNVTPHLLAGMR(1)-QAKEFTK(3)	1.17E-03	1
Set1(799)-Cps40(130)	QLLSTYTPTVTPETSAALEYKIWQSR(21)-LKTDEDR(2)	1.44E-03	3
Cps25(124)-Set1(425)	KYLNTNVTPHLLAGMRLIAVQQPEDPLR(1)-PVLHVSKIFVAKHR(7)	2.63E-03	1
Cps15(56)-Set1(164)	VASTVKEMVNEDEELIFK(6)-DKMLSWKATDK(2)	4.68E-03	1
Cps15(68)-Set1(576)	EMVNEDEELIFKNR(12)-ELKEK(3)	4.96E-03	4
Cps60(17)-Cps25(107)	LGIIPYQEGTDIVYKNALQGQQEGK(15)-ISKLENVNLAATVGGSQTR(3)	1.15E-02	2
Set1(224)-Cps35(8)	VATHRKCR(6)-MTTVSINK(8)	3.36E-02	3
Set1(164)-Cps40(96)	DKMLSWK(2)-RKCR(2)	4.24E-02	1
Set1(594)-Cps35(15)	IAEDELKR(7)-FKHVK(2)	5.35E-02	1

Set1(273)-Cps30(184)	NYFKK(4)-DWKR(3)	5.92E-02	2
Set1(203)-Cps50(109)	HRHPEIKASDPR(7)-PLKEIR(3)	6.46E-02	1
Set1(986)-Cps30(184)	YLKNGIGSSYLFR(3)-DWKR(3)	7.30E-02	1
Set1(1007)-Cps30(181)	VDENTVIDATKK(11)-TLTYDKDWKR(6)	8.05E-02	5
Set1(224)-Cps15(14)	VATHRKCR(6)-RLSDKYK(5)	8.08E-02	1
Set1(982)-Cps50(109)	QPVAEMREKR(9)-PLKEIR(3)	9.55E-02	1
Cps15(9)-Set1(357)	MAYNQEDSKR(9)-KLQK(1)	1.15E-01	1
Cps30(175)-Set1(441)	IFDAETGHCLKTLTYDK(11)-YKLR(2)	1.38E-01	4
Cps30(121)-Set1(194)	GNLLFTSSMDESIKIWDTLNGSLMKTISAHSEAVVSVDVPMNDSSILSSGSYDGL IR(14)-ELQDGQFKFKIK(10)	1.51E-01	1
Cps50(18)-Set1(587)	EHPEKLTHTIENPLR(5)-QQIASKIAEDELK(6)	1.63E-01	1
Cps40(260)-Cps35(1)	KNICGYCSTYER(1)-MTTVSINK(1)	3.11E-01	1
Set1(1008)-Cps60(285)	KGGIAR(1)-KKLK(2)	4.33E-01	1
Cps40(96)-Set1(1008)	KCRISDCYK(1)-KGGIAR(1)	6.24E-01	1
Cps35(68)-Set1(637)	FLDTIASKK(8)-KKLSNGIK(2)	6.37E-01	1
Cps30(273)-Cps35(146)	GVLHHSCGMDFLNPEDGSTPLVISGYENGDIYCWNSDTKSLLQLLDGSLYHHSS PVMSIHCFGNIMCSLALNGDCCLWR(39)-ISKPQVIIPSLVPNCIAYDPSGLVFAL GNPENFEIGLYNLK(3)	6.80E-01	1
Cps60(293)-Cps50(106)	QETTNKEFQR(6)-PSKPLK(3)	6.83E-01	1

* Our XL-MS analysis also indicated that although we did not include the Cps15 in our plasmid, still, in our purified COMPASS, Cps15 doesexist and involves in the interaction with Set1 subunit.

Table S4. List of the plasmids used in this study

Plasmid	Cloning vector	Insert
IV1	pRS303	*GAL4-GAL1-10-TAP-SET1*
IV2	pRS304	*CPS60-GAL1-10-CPS50*
IV3	pRS305	*CPS40-GAL1-10-CPS35*
IV4	pRS306	*CPS30-GAL1-10-CPS25*
IV1-D	pRS303	*GAL4-GAL1-10-TAP-DID1-SET1*
IV2-60E	pRS304	*CPS60-eGFP-GAL1-10-CPS50*
IV2-50PA	pRS304	*CPS60-GAL1-10-CPS50(134PA)*
pET28a-Dyn2	pET28a	*6 × His-rTEV-DYN2*
pET28a-DID2	pET28a	*GST- rTEV-DID2*

Table S5. List of *S. cerevisiae* strains used in this study

Name of strain	Genotype of strain
W303a	*MATa, ura3-1, can1-100, leu2-3,112, trp1-1,ade2-1,his3-11*
yCOS1	*W303a pep4::KanMx4*
yCOS2	*W303a pep4::KanMx4, his3-11::HIS3IV1,CPS60::CPS60IV2, CPS35::CPS3IV3, CPS30::CPS30IV4*
yCOS3	*W303a pep4::KanMx4, dyn2::Hph-NT1*
yCOS4	*W303a pep4::KanMx4, dyn2::Hph-NT1, his3-11::HIS3IV1-D, CPS60::CPS60IV2, CPS35::CPS35IV3, CPS30::CPS30IV4*
yCOS5	*W303a pep4::KanMx4, CPS60::Hph-NT1*
yCOS6	*W303a pep4::KanMx4, CPS50::Hph-NT1*
yCOS9	*W303a pep4::KanMx4, CPS60::Hph-NT1, his3-11::HIS3IV1-D, CPS50::CPS50IV2-60E, CPS35::CPS35IV3, CPS30::CPS30IV4*
yCOS11	*W303a pep4::KanMx4, CPS50::Hph-NT1, his3-11::HIS3IV1-D, CPS60::CPS60IV2-50PA, CPS35::CPS35IV3, CPS30::CPS30IV4*

Full-length gel and blots presented in figure 1

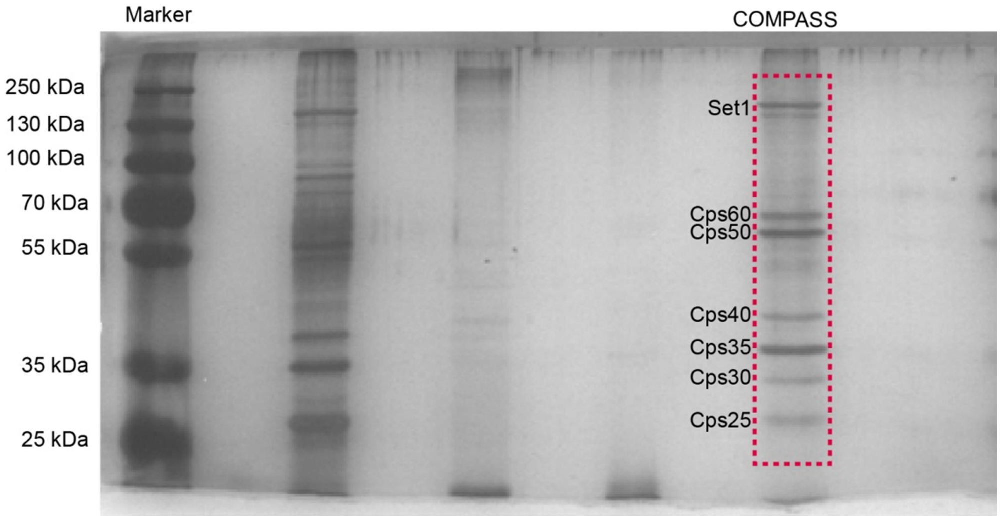

The original gel of Figure 1B. SDS-PAGE analysis of the purified COMPASS complex. 10% SDS-PAGE was stained by silver. PageRuler plus prestained ladder is used as marker. Information within the red frame was presented in Fig.1B

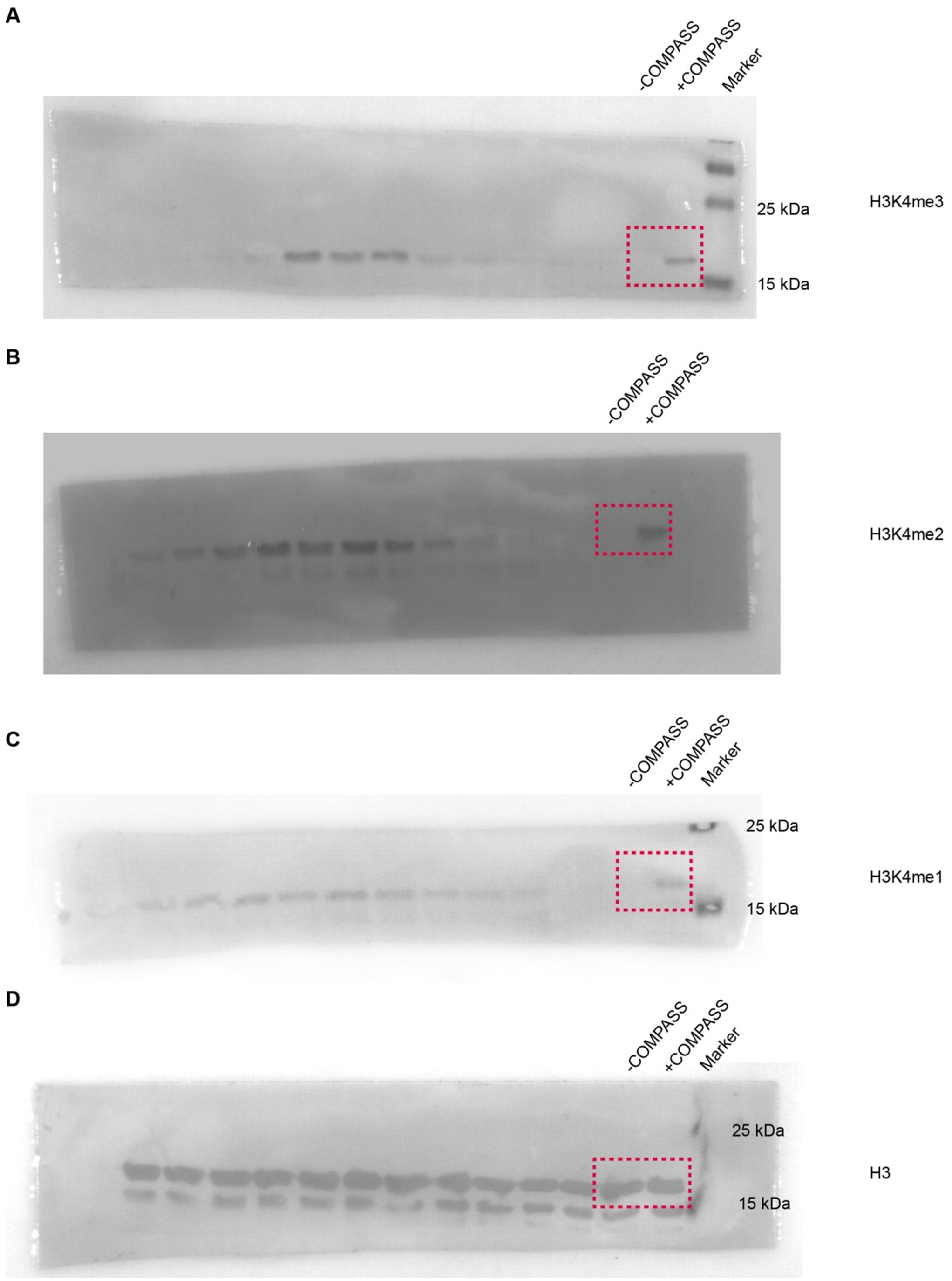

The original western-blots of figure 1C. In vitro H3K4 methyltransferase analysis. (A-C) In vitro H3K4 mono-, di-, and tri-methylation activities of COMPASS complex, examined by western blotting using H3K4me1, me2, and me3 antibody, respectively. (D) Anti-H3 antibody was used as control. Two bands were visualized here, which may be caused by the degradation of histone H3. Other lanes were used to analyze the H3K4 methyltransferase activity of the gradient centrifugation fractions. Information within the red frame was presented in Fig.1C.
